# Consumption of coffee associated with reduced risk of liver cancer: a meta-analysis

**DOI:** 10.1186/1471-230X-13-34

**Published:** 2013-02-25

**Authors:** Li-Xuan Sang, Bing Chang, Xiao-Hang Li, Min Jiang

**Affiliations:** 1 Department of Cadre Ward II, First Affiliated Hospital of China Medical University, No.155, Nanjing North Street, Heping District, 110001, Liaoning Province, Shenyang, China; 2 Department of Gastroenterology, First Affiliated Hospital of China Medical University, No.155, Nanjing North Street, Heping District, 110001, Liaoning Province, Shenyang, China; 3 Department of General Surgery, First Affiliated Hospital of China Medical University, No.155, Nanjing North Street, Heping District, 110001, Liaoning Province, Shenyang, China

**Keywords:** Coffee, Epidemiology, Liver cancer, Meta-analysis

## Abstract

**Background:**

Epidemiologic studies have reported inconsistent results regarding coffee consumption and the risk of liver cancer. We performed a meta-analysis of published case–control and cohort studies to investigate the association between coffee consumption and liver cancer.

**Methods:**

We searched Medline, EMBASE, ISI Web of Science and the Cochrane library for studies published up to May 2012. We performed a meta-analysis of nine case–control studies and seven cohort studies.

**Results:**

The summary odds ratio (OR) for high *vs* no/almost never drinkers was 0.50 (95% confidence interval (CI): 0.42–0.59), with no significant heterogeneity across studies (Q = 16.71; *P* = 0.337; I^2^ = 10.2%). The ORs were 0.50 (95% CI: 0.40–0.63) for case–control studies and 0.48 (95% CI: 0.38–0.62) for cohort studies. The OR was 0.38 (95% CI: 0.25–0.56) in males and 0.60 (95% CI: 0.33–1.10) in females. The OR was 0.45 (95% CI: 0.36–0.56) in Asian studies and 0.57 (95% CI: 0.44–0.75) in European studies. The OR was 0.39 (95% CI: 0.28–0.54) with no adjustment for a history of liver disease and 0.54 (95% CI: 0.46–0.66) after adjustment for a history of liver disease.

**Conclusions:**

The results of this meta-analysis suggested an inverse association between coffee consumption and liver cancer. Because of the small number of studies, further prospective studies are needed.

## Background

Primary liver cancer is a common malignancy worldwide. It is the fifth most common cancer in men and the cause of a third of male cancer deaths. It is the eighth most common cancer in women and the sixth most common cause of female cancer deaths [1]. Chronic infection with hepatitis B or C viruses and alcohol consumption are considered the most important risk factors for liver cancer [2-5]. A large number of epidemiological studies indicated that environmental factors can affect the risk of liver cancer, but the role of dietary factors in tumorigenesis has not yet been determined [6-8]. Analysis of environmental factors that may be associated with liver cancer has become a popular research topic in recent years.

Coffee contains many biologically active components, some of which may have anti-tumor effects. Epidemiological studies have reported inconsistent findings on the association between coffee and liver cancer. We therefore carried out a meta-analysis of prospective cohort studies and case–control studies in order to clarify the association between coffee consumption and liver cancer.

## Methods

### Search strategy

We searched Medline (via PubMed; National Library of Medicine), EMBASE (Elsevier, Amsterdam, the Netherlands), ISI Web of Science (Institute for Scientific Information, Philadelphia, Pennsylvania), and the Cochrane library (Wiley, Chichester, United Kingdom) for studies published up to May 2012. Key words searched were as follows: (coffee OR caffeine OR beverages OR diet OR drinking OR lifestyle) AND (liver OR hepatocellular OR digestive) AND (cancer OR carcinoma OR tumor OR neoplasm) AND (risk). No language restrictions were applied.

### Inclusion and exclusion criteria

The inclusion criteria were: case–control or cohort study; data on the frequency of coffee consumption; primary outcome defined as liver cancer or hepatocellular carcinoma; and relative risk (RR) estimates, odds ratios (ORs) or hazard ratios (HRs) with their corresponding 95% confidence intervals (CIs). Exclusion criteria included duplicate reports and insufficient data about coffee consumption.

### Data extraction

The following data were collected from each publication: the name of the first author, year of publication, the country where the study was conducted, sex, study design, study population demographics, study period, sample size, type of outcome, consumption of coffee, number of exposed cases, the RRs or ORs or HRs and their 95%CIs, and covariates adjusted in the analysis. All data were extracted independently by three reviewers, and any disagreement was resolved by discussion between them. If results were published more than once, the results from the most recent one were selected. Because liver cancer is rare, the OR was assumed to be the same as RR and HR, and all results are reported as OR for simplicity [9].

### Quality assessment

The study quality was assessed by the 9-star Newcastle-Ottawa Scale [10]. A full score is 9 stars, and a score ≥ 6 stars is considered to be high quality. The quality of case–control studies was assessed as follows: adequate definition of cases, representativeness of cases, selection of controls, definition of controls, control for the most important factor or the second important factor, exposure assessment, same method of ascertainment for all subjects, and non-response rate (Table 1). The quality of cohort studies was assessed as follows: representativeness of the exposed cohort, selection of the unexposed cohort, ascertainment of exposure, outcome of interest not present at start of study, control for the most important factor or the second important factor, outcome assessment, follow-up long enough for outcomes to occur, adequacy of follow-up of cohorts (Table 2).

**Table 1:** **Quality assessment of case–control studies included in this meta-analysis**^**1**^

**Study**	**Adequate definition of cases**	**Representativeness of cases**	**Selection of control**	**Definition of control**	**Control for important factor or additional factor**^**2**^	**Exposure assessment**	**Same method of ascertainment for cases and controls**	**Nonresponse rate**^**3**^	**Total quality scores**
Kuper et al. [11], 2000	★	★	-	★	★	-	★	-	5
Gallus et al. [12], 2002	★	★	-	★	★	-	★	-	6
Gelatti et al. [13], 2005	★	★	-	★	★★	★	★	-	7
Ohfuji et al. [14], 2006	★	★	-	★	★★	-	★	-	6
Tanaka et al. [15], 2007	★	★	★	★	★	-	★	-	6
Montella et al. [16], 2007	★	★	-	★	★★	-	★	-	6
Wakai et al. [17], 2007	★	★	★	★	★	-	★	-	6
Ohish et al. [18], 2008	★	★	★	★	★★	-	★	-	7
Leung et al. [19], 2011	★	★	-	★	-	★	★	-	5

^1^A study can be awarded a maximum of one star for each numbered item except for the item Control for most important factor or second important factor.

^2^ A maximum of two stars can be awarded for Control for most important factor or second important factor. Studies that controlled for hepatitis B virus (HBV) or HCV infection received one star, whereas studies that controlled for alcohol drinking received one additional star.

^3^ One star was awarded if there was no significant difference in the response rate between control subjects and cases in the chi-square test (*P* > 0.05).

**Table 2:** Q**uality assessment of cohort studies included in this meta- analysis**^**1**^

**Study**	**Representativeness of the exposed cohort**	**Selection of the unexposed cohort**	**Ascertainment of exposure**	**Outcome of interest not present at start of study**	**Control for important factor or additional factor**^**2**^	**Outcome assessment**	**Follow-up long enough for outcomes to occur**^**3**^	**Adequacy of follow-up of cohorts**^**4**^	**Total quality scores**
Shimazu et al. [20], 2005	★	★	★	★	★★	★	★	★	9
Shimazu et al. [20], 2005	★	★	★	★	★★	★	★	★	9
Inoue et al. [21], 2005	-	★	★	★	★	★	★	★	7
Kurozawa et al. [22], 2005	★	★	★	★	★★	★	★	-	8
Hu et al. [23], 2008	★	★	★	★	★★	★	★	★	9
Inoue et al. [24], 2009	-	★	★	★	★★	★	★	★	8
Johnson et al. [25], 2011	-	★	★	★	★	★	★	★	7

^1^A study can be awarded a maximum of one star for each numbered item except for the item Control for most important factor or second important factor.

^2^ A maximum of two stars can be awarded for Control for most important factor or second important factor. Studies that controlled for HBV or HCV infection received one star, whereas studies that controlled for alcohol drinking received one additional star.

^3^ A cohort study with a follow-up time > 7 y was awarded one star.

^4^ A cohort study with a follow-up rate > 75% was awarded one star.

### Statistical analysis

For the included studies, we determined pooled ORs (or RRs or HRs) with 95% CI for the highest versus lowest category of coffee consumption from each study. Since various sources of heterogeneity may exist owing to a variety of factors, we carried out subgroup analysis to investigate the influence of study design, study region, sex and history of liver disease on the heterogeneity.

Statistical heterogeneity was evaluated through the Q test and I^2^ statistic [26]; *P* < 0.10 was considered statistically significant [27]. If the heterogeneity was acceptable (I^2^ < 50%), a fixed effects analysis was conducted to calculate the pooled OR. In addition, a random effects model was used. The causes of heterogeneity were investigated by subgroup analyses. To evaluate whether publication bias might affect the statistical results, we applied Egger’s test and Begg’s method to assess bias through visual inspection of funnel plots [28,29]; all statistical analyses were conducted using STATA (version 11.0; StataCorp, College Station, TX, USA). All statistical tests were 2-sided.

## Results

### Study characteristics

**Figure 1:**
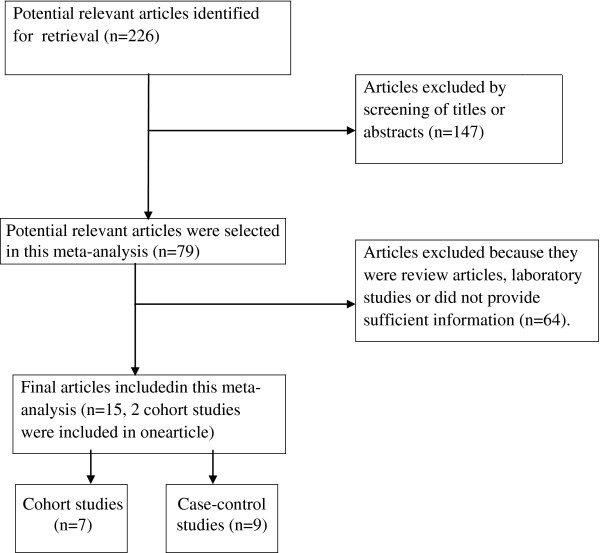
Process of study selection in the meta-analysis.

Figure 1 shows the process of selecting studies for the meta-analysis. Sixteen observational articles examining the association between coffee consumption and the risk of liver cancer were included in our meta-analysis (Table 3) [11-25]. There were nine case–control studies [11-19] and seven cohort studies (two of these were nested in a cohort article) [20-25]. Of the selected studies, 11 were conducted in Asia (nine in Japan [14,15,17,18,20-22,24], one in Singapore [25], one in Hong Kong [19]) and five in Europe (one in Finland [23], two in Italy [13,16], one in Greece [11], one in Italy and Greece [12]). Among case–control studies, seven were hospital-based case–control studies [11-16,19], and two were nested case- control studies [17,18].

**Table 3:** Characteristics of studies included in the meta-analysis

**Author**	**Design**	**Study population**	**Study period and outcome**	**Case/Control**	**Coffee consumption**	**Risk estimate (95% CI)**	**Covariate adjustments**
Kuper et al. [11], 2000	HCCS	Greece	1995–1998 HCC incidence	333/360	Nondrinkers	1	Age, gender, years of schooling, HBsAg and/or anti-HCV status
					<20 cups/week	1.1 (0.5–2.6)	
					≥20 cups/week	0.9 (0.4–2.5)	
Gallus et al. [12], 2002	HCCS	Greece Italy	1995–1998	834/1912	Nondrinkers	1.0	Age, sex, smoking, alcohol drinking, history of diabetes or hepatitis, education, BMI
			1984–1997 HCC incidence		1 cup/day	1.2 (0.9–1.6)	
					2 cup/day	1.0 (0.7–1.3)	
					≥3 cups/day	0.7 (0.5–1.0)	
Shimazu et al. [20], 2005	CS1	Japan	1984–1992 Primary liver cancer incidence	70/22404	Nondrinkers	1.0	Age, sex, smoking, alcohol drinking, history of liver disease
					occasionally	0.56 (0.33–0.97)	
					≥1 cups/day	0.53 (0.28–1.00)	
Shimazu et al. [20], 2005	CS2	Japan	1990–1997 Primary liver cancer incidence	47/38703	Nondrinkers	1.0	Age, sex, smoking, alcohol drinking, history of liver disease
					occasionally	1.05 (0.52~2.16)	
					≥1 cups/day	0.68 (0.31~1.51)	
Kurozawa et al. [22], 2005	CS	Japan	1988–1999 HCC mortality	258/83966	Total		Age, sex, smoking, alcohol habits, history of diabetes or liver disease, education
					Nondrinkers	1.0	
					<1 cup/day	0.83 (0.54–1.25)	
					≥1 cup/day	0.50 (0.31–0.79)	
					Men		
					Nondrinkers	1.0	
					<1 cup/day	0.91(0.57–1.45)	
					≥1 cup/day	0.49(0.28–0.85)	
					Women		
					Nondrinkers	1.0	
					<1 cup/day	0.64 (0.27–1.51)	
					≥1 cup/day	0.51 (0.20–1.31)	
Inoue et al. [21], 2005	CS	Japan	1990–2001 HCC incidence	334/90452	Men and women		Age, sex, study area, ethanol intake, green vegetable, green tea and smoking
					Almost never	1.0	
					1–2 day/week	0.75 (0.56–1.01)	
					3–4 day/week	0.79 (0.55–1.14)	
					1–2 cups/day	0.52 (0.38–0.73)	
					3–4 cups/day	0.48 (0.28–0.83)	
					≥5 cups/day	0.24 (0.08–0.77)	
					Men		
					Almost never	1.0	
					1–2 day/week	0.74 (0.52–1.05)	
					3–4 day/week	0.76 (0.50–1.16)	
					1–2 cups/day	0.55 (0.38–0.80)	
					3–4 cups/day	0.41 (0.21–0.77)	
					≥5 cups/day	0.27 (0.09–0.87)	
					women		
					Almost never	1.0	
					1–2 day/week	0.77 (0.43–1.37)	
					3–4 day/week	0.89 (0.43–1.84)	
					1–2 cups/day	0.43 (0.20–0.90)	
					3–4 cups/day	0.89 (0.31–2.59)	
					≥5 cups/day	-----	
Gelatti et al. [13], 2005	HCCS	Italy	1994–2003 HCC incidence	250/500	Nondrinkers	1.0	Age, sex, alcohol drinking, HBV and/or HCV infection
					1–2 cups/day	0.8 (0.4–1.3)	
					3–4 cups/day	0.4 (0.2–0.8)	
					≥5 cups/day	0.3 (0.1–0.7)	
Ohfuji et al. [14], 2006	HCCS	Japan	2001–2002 HCC incidence	73/253	Nondrinkers	1.0	Age, sex, smoking, alcohol drinking, time since first identification of liver disease, BMI, disease severity, family history of liver disease, interferon therapy
					<1 cup/day	0.61 (0.18–2.03)	
					≥1 cup/day	0.38 (0.13–1.12)	
Tanaka et al. [15], 2007	PCCS	Japan	2001–2004 HCC incidence	209/1253	Nondrinkers	1.0	Age, sex, smoking status, heavy alcohol drinking,
					occasionally	0.33 (0.22~0.48)	
					1–2 cups/day	0.27 (0.15~0.48)	
					≥3 cups/day	0.22 (0.11~0.43)	
Montella et al. [16], 2007	HCCS	Italy	1999–2002 HCC incidence	185/412	Abstainers	2.28 (0.99–5.24)	Age, sex, alcohol drinking, HBV and/or HCV infection, education, smoking, alcohol drinking
					<14 cups/week	1.0	
					14–20 cups/week	0.54 (0.27–1.07)	
					21–27 cups/week	0.57 (0.25–1.32)	
					≥28 cups/week	0.43 (0.16–1.13)	
Wakai et al. [17], 2007	NCCS	Japan	1988–1990 HCC incidence	96/3444	Nondrinkers	1.0	Age, sex, smoking, alcohol drinking, consumption of areca,educational levels, ethnicity,source of hospital
					<1 cup/day	0.77 (0.45–1.32)	
					≥1 cup/day	0.49 (0.25–0.96)	
Hu et al. [23], 2008	CS	Finland	1997–2002 HCC incidence	128/60323	Total		Age, sex, smoking, alcohol drinking, education, study year, diabetes and chronic liver disease BMI and during follow up.
					0–1 cup/day	1.0	
					2–3 cups/day	0.66 (0.37–1.16)	
					4–5 cups/day	0.44 (0.25–0.77)	
					6–7 cups/day	0.38 (0.21–0.69)	
					≥8 cups/day	0.32 (0.16~0.62)	
					Men		
					0–1 cup/day	1.0	
					2–3 cups/day	0.68 (0.35–1.31)	
					4–5 cups/day	0.35 (0.18–0.71)	
					6–7 cups/day	0.31 (0.15–0.63)	
					≥8 cups/day	0.28 (0.13–0.61)	
					Women		
					0–1 cup/day	1.0	
					2–3 cups/day	0.62 (0.19–2.04)	
					4–5 cups/day	0.60 (0.20–1.82)	
					6–7 cups/day	0.58 (0.19–1.82)	
					≥8 cups/day	0.41 (0.10–1.70)	
Ohishi et al. [18], 2008	NCCS	Japan	1999–2002 HCC incidence	224/644	Nondrinkers	1.0	Hepatitis virus infection, alcohol consumption, smoking habits, BMI, diabetes mellitus, and radiation dose to the liver
					Daily	0.40 (0.16–1.02)	
Inoue et al. [24], 2009	CS	Japan	1993–1994 HCC incidence	110/18815	Total		Age, sex, area, smoking, alcohol drinking, BMI, diabetes mellitus, green tea consumption, serum ALTlevel, and HBV and HCV infection status
					Almost never	1.0	
					<1 cup/day	0.67 (0.42–1.07)	
					1–2 cups/day	0.49 (0.27–0.91)	
					≥3 cups/day	0.54 (0.21–1.39)	
					Men		
					Almost never	1.0	
					<1 cup/day	0.79 (0.46–1.37)	
					1–2 cups/day	0.37 (0.17–0.81)	
					≥3 cups/day	0.32 (0.10–1.10)	
					Women		
					Almost never	1.0	
					<1 cup/day	0.39 (0.15–1.03)	
					1–2 cups/day	0.92 (0.36–2.38)	
					≥3 cups/day	0.69 (0.11–4.22)	
Johnson et al. [25], 2011	CS	Chinese	1993–2006 HCC incidence	362/61321	Nondrinkers	1.0	Age at recruitment, sex, dialect group, year of recruitment, BMI, level of education, consumption of alcoholic beverages, smoking, black tea and green tea intake, and history of diabetes.
					0-<1 drinkers/day	0.94 (0.63–1.40)	
					1-<2 drinkers/day	1.17 (0.87–1.56)	
					2-<3 drinkers/day	0.78 (0.56–1.07)	
					≥3 drinkers/day	0.56 (0.31–1.00)	
Leung et al. [19], 2011	HCCS	HongKong	2007–2008 HCC incidence	109/125	<1 time/week	1.0	Age, sex, alcohol drinking, cigarette smoking, tea consumption and physical activity
					1–3 times/week	0.58 (0.24–1.36)	
					≥4 times/week	0.41 (0.19–0.89)	

ALT: alanine aminotransferase; BMI: body mass index; CI: confidence interval; HbsAg: hepatitis B surface antigen; HCCS: hospital-based case–control study; PCCS: population-based case–control study; NCCS: nested case–control study; CS: cohort study; HCC: hepatocellular carcinoma.

### High *vs* Non/Almost never drinkers

**Figure 2:**
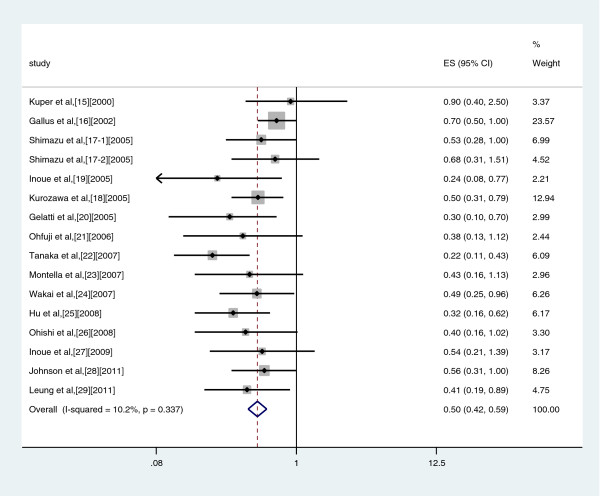
Risk estimates from studies assessing the association between high coffee consumption (highest versus non/occasionally) and liver cancer risk.

**Figure 3:**
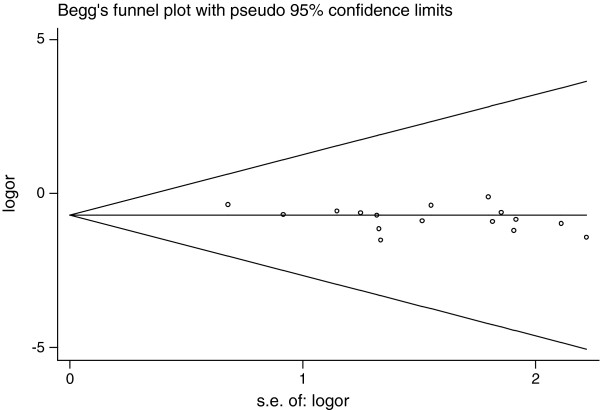
Begg’s funnel plot of coffee consumption and risk of liver cancer.

A meta-analysis of risk estimates for the incidence of liver cancer for highest compared with lowest coffee consumption categories could be conducted with data from nine case–control studies and nine cohort studies. Our results showed a 50% reduction in risk of liver cancer with the highest intake of coffee (summary OR: 0.50, 95%CI: 0.42–0.59) (Figure 2). There was no significant heterogeneity across studies (Q = 16.71, *P* = 0.337, I^2^ = 10.2%). There was a symmetric funnel plot and no evidence of significant publication bias from Egger’s test (*P* = 0.05) and Begg’s test (*P* = 0.096) (Figure 3).

A sensitivity analysis for the risk of liver cancer was performed by excluding one study [22], the outcome of which was mortality. The summary OR was 0.49 (95%CI: 0.41–0.59). There was no significant heterogeneity across studies (Q = 16.7, *P* = 0.272, I^2^ = 16.2%).

**Figure 4:**
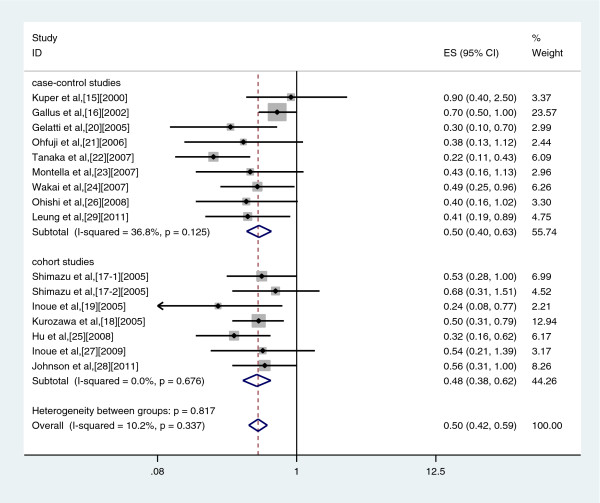
Forest plot of coffee consumption and risk of liver cancer, stratified by study type.

Similar results were found in a subgroup analyses conducted by study design in case–control studies (OR: 0.50, 95%CI: 0.40–0.63, Q = 12.38, *P* = 0.125, I^2^ = 36.8%), and cohort studies (OR: 0.48, 95% CI: 0.38–0.62, Q = 2.47, *P* = 0.676, I^2^ = 0.0%) (Figure 4).

In a subgroup analysis conducted by sex, only four studies were included in the analysis: studies in males gave an OR of 0.38 (95% CI: 0.25–0.56, Q = 1.83, *P* = 0.609, I^2^ = 0.0%), while studies in females gave an OR of 0.60 (95% CI: 0.33–1.10, Q = 0.94, *P* = 0.815, I^2^ = 0.0%) (Table 4).

**Table 4:** Pooled relative risks and 95% CI for coffee consumption and liver cancer risk

**Study**	**No. of studies**	**No. of cases**	**Relative risk (95% CI)**	** Heterogeneity**		
				**Q**	***P***	**I**^**2**^**(%)**
High versus non/almost never intake						
All studies	16	3,622	0.50 (0.42–0.59)	16.71	0.337	10.2%
Study design						
Cohort studies	7	1,309	0.48 (0.38–0.62)	2.47	0.676	0.0%
Case–control studies	9	2,313	0.50 (0.40–0.63)	12.38	0.125	36.8%
Study region						
Asia	11	1,892	0.45 (0.36–0.56)	7.86	0.642	0.0%
Europe	5	1,730	0.57 (0.44–0.75)	7.09	0.131	43.6%
Study gender						
Male	4	583	0.38 (0.25–0.56)	1.83	0.609	0.0%
Female	4	247	0.60 (0.33–1.10)	0.94	0.815	0.0%
Adjustment for main confounders^a^						
Adjusted	11	2,512	0.54 (0.46–0.66)	8.5	0.581	0.0%
Unadjusted	5	1,110	0.39 (0.28–0.54)	5.34	0.254	25.1%

^a^main confounder: hepatitis B and hepatitis C virus infection or history of liver disease.

**Figure 5:**
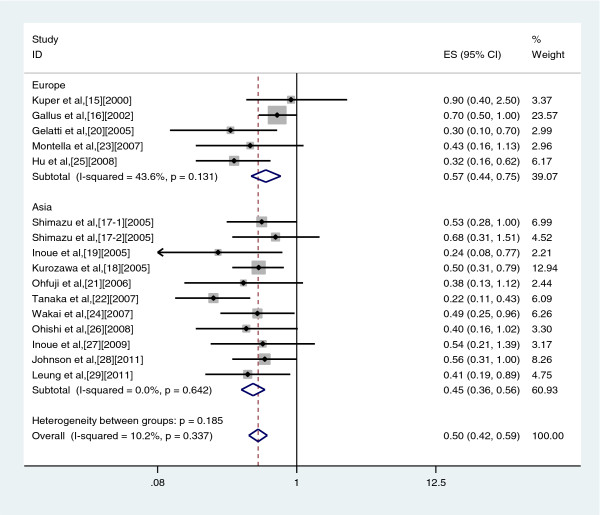
Forest plot of coffee consumption and risk of liver cancer, stratified by study region.

When stratified analysis was conducted by study region, a statistically significant protective effect of coffee consumption on liver cancer was observed in Asia (OR: 0.45, 95% CI: 0.36–0.56, Q = 7.86, *P* = 0.642, I^2^ = 0.0%), and in Europe (OR: 0.57, 95% CI: 0.44–0.75, Q = 7.09, *P* = 0.131, I^2^ = 43.6%) (Figure 5).

Stratification analysis was conducted without or with adjustment for a history of liver disease. A statistically significant protective effect of coffee consumption on liver cancer was observed with no adjustment for a history of liver disease (OR: 0.39, 95% CI: 0.28–0.54, Q = 5.34, *P* = 0.254, I^2^ = 25.1%) and after adjustment for a history of liver disease (OR: 0.54, 95% CI: 0.46–0.66, Q = 8.5, P = 0.581, I^2^ = 0.0%).

## Discussion

Coffee consumption has been suggested as a protective factor in the development of liver cancer, but evidence from observational studies is inconsistent [11-25]. The results of the current meta-analysis of seven prospective and nine case–control studies suggest that there is an inverse association between coffee consumption and liver cancer among different groups according to consumption level. There were significant reductions of 50% in the risk of liver cancer with the highest consumption of coffee compared with non/almost never consumption. The meta-analyses of Bravi et al. [30] found significant reductions of 55% in the risk of liver cancer with the high drinkers compared with non-drinkers, and Larsson & wolk [31] found a risk reduction of 43% per 2 cups of coffee per day increment. Our results are consistent with these two previous articles, partly because all of the studies in these two articles are included in the our meta-analysis.

Some results in this meta-analysis were heterogeneous, because the included studies had differences in study design, study region, study sex distribution, and control for confounding factors. In separate analyses by study design, we found an inverse association between coffee consumption and liver cancer among hospital- based case–control studies and among cohort studies.

There was also an inverse association between coffee consumption and liver cancer among European and Asian populations, and the significant risk reduction was stronger among Asian than European populations. The different results may be explained by racial differences. Differences in coffee drinking habits may be a partial explanation for the discrepancy.

We also found an inverse association between coffee consumption and liver cancer among male and female populations, but this result was derived from only four studies with a small number of cases, so we could not draw a firm conclusion. A history of liver disease may be a risk factor for liver cancer, and after adjustment for this, a significant inverse association remained between coffee consumption and liver cancer among two subgroups.

There are several potential mechanisms through which high consumption of coffee may reduce the risk of liver cancer. Coffee contains a variety of chemicals including caffeine, cafestol, kahweol, and chlorogenic acids. It remains uncertain which ingredient of coffee is protective against liver cancer. Some studies have indicated that caffeine can prevent oxidative DNA damage, modify the apoptotic response and reverse cell cycle checkpoint function [32-34]. Caffeine has strong antioxidant properties [35]. In an animal experiment, caffeine significantly reduced the incidence of chemically-induced hepatocellular carcinoma in rats [36]. Furthermore, cafestol and kahweol have been shown to be anti-carcinogenic [37,38]. Cafestol and kahweol have demonstrated a protective effect against aflatoxin B1-induced genotoxicity [39]. In addition, a study by Feng et al. showed that chlorogenic acids can scavenge reactive oxygen species and have an anti-tumor effect [40]. These studies suggest that ingredients in coffee may play an important role in protecting against the occurrence and development of liver cancer.

Our meta-analysis had some merits. First, the total number of cases included in this meta-analysis was substantial (n = 3622 liver cancer cases). The summary ORs of the highest compared with the lowest coffee consumption categories for risk of liver cancer were consistent with those in a previously published meta-analyses (n = 2260 liver cancer cases) [30,31]. Second, we found little evidence of publication bias in our meta-analysis. Third, we performed a comprehensive search of the literature on the association between coffee consumption and liver cancer risk up to May 2012.

Our meta-analysis had several limitations. First, we used the highest and lowest coffee consumption levels as measures of exposure, but we were not able to determine whether different amounts of coffee consumption could decrease liver cancer risk. Second, misclassification bias should be considered. Each study presented coffee consumption in different units (cups/week, cups/day, days/week, drinks/day, times/week). Therefore, differential misclassification could bias the results. Third, because liver cancer is a multifactorial disease, it is uncertain whether other factors may have influenced the results. Fourth, the study areas covered in our meta-analysis only included Asia (Japan, China, Hong Kong) and Europe (Finland, Greece, Italy). Therefore, the value of our results is limited for other areas (Africa, America and Australia). Fifth, potential publication bias might have influenced the results, despite no bias indicated from either the funnel plot or Egger’s test.

## Conclusion

The results of this meta-analysis suggested that coffee consumption may be associated with a reduced risk of liver cancer. However, because of potential confounding, this finding should be treated with caution. Further better-controlled studies are needed to confirm this finding.

## Competing interests

The authors declare no potential conflict of interest relevant to this research.

## Authors’ contributions

LXS and BC designed the research; LXS and XHL performed the literature search; LXS, XHL and MJ analyzed the data and interpreted the results; LXS and BC wrote the paper; all authors approved the final manuscript.

## Pre-publication history

The pre-publication history for this paper can be accessed here:

http://www.biomedcentral.com/1471-230X/13/34/prepub
